# Excited Delirium and Sudden Death: A Syndromal Disorder at the Extreme End of the Neuropsychiatric Continuum

**DOI:** 10.3389/fphys.2016.00435

**Published:** 2016-10-13

**Authors:** Deborah C. Mash

**Affiliations:** Department of Neurology and Molecular and Cellular Pharmacology, University of Miami Miller School of MedicineMiami, FL, USA

**Keywords:** delirium, CNS, neurocardiac, dopamine, dopamine transporter, mania, cocaine

## Abstract

Over the past decade, the excited delirium syndrome (ExDS) has raised continued controversy regarding the cause and manner of death of some highly agitated persons held in police custody, restrained or incapacitated by electrical devices. At autopsy, medical examiners have difficulty in identifying an anatomic cause of death, but frequently cite psychostimulant intoxication as a contributing factor. The characteristic symptoms of ExDS include bizarre and aggressive behavior, shouting, paranoia, panic, violence toward others, unexpected physical strength, and hyperthermia. Throughout the United States and Canada, these cases are most frequently associated with cocaine, methamphetamine, and designer cathinone abuse. Acute exhaustive mania and sudden death presents with behavioral symptoms that are identical to what is described for ExDS in psychostimulant abusers. Bell's mania or acute exhaustive mania was first described in the 1850's by American psychiatrist Luther Bell in institutionalized psychiatric patients. This rare disorder of violent mania, elevated body temperature and autonomic collapse continued to be described by others in the psychiatric literature, but with different names until the first cases of ExDS were seen at the beginning of the cocaine epidemic by medical examiners. The neurochemical pathology examination of brain tissues after death revealed a loss of dopamine transporter regulation together with increases in heat shock protein 70 (hsp70) expression as a biomarker of hyperthermia. The similarity in the behavioral symptoms between extremely agitated psychostimulant abusers and unmedicated psychiatric patients suggests that a genetic disorder that leads to dysregulated central dopamine transporter function could be a precipitating cause of the acute delirium and sudden death. While the precise cause and mechanism of lethality remains controversial, the likely whys and wherefores of sudden death of ExDS victims are seen to be “biological,” since excessive dopamine in the brain triggers the manic excitement and delirium, which unabated, culminates in a loss of autonomic function that progresses to cardiorespiratory collapse.

## Inset

Henry Maudsley MD described Acute Mania and Acute Maniacal Delirium in 1867 in his “Physiology and Pathology of the Mind,” which best illustrates the view discussed in this article. He suggests that persons in an agitated state of acute mania benefit from “abundant exercise in the open air” while “such a practice would be most unscientific in acute delirium, and very likely to be followed by fatal consequences”. He further states “it would be better to place a patient suffering from such acute degeneration of cerebral function entirely in seclusion” rather “than to aggravate his disorder by forced exercise and mischievous struggles with attendants”. Medico-legal reports more than a hundred and fifty years after Maudsley and Luther Bell find the prognosis is never very favorable for individuals at risk for excited delirium.

## Historical descriptions and case reports

Psychiatrists in the United Kingdom, France and America were the first to provide clinical descriptions and case reports of persons in states of acute exhaustive mania and delirium. In the 1800s, Dr. Luther Bell, psychiatrist at the McLean Asylum for the Insane in Massachusetts described a clinical condition with a 75 percent mortality rate. “Bell's mania” or acute exhaustive mania was characterized by delusions, hallucinations, hyperactivity, and frequent fevers. The descriptions although similar to the psychotic features of paranoid schizophrenics (e.g., hallucinations and delusions) revealed a more extreme condition of generalized severe disorganization of behavior, including hyperactive arousal, altered sleep-wake cycle, and elevated core body temperature. Calmeil's report of an uncommon, but life threating psychosis with extreme hyperactivity and mounting fear fading to stuperous exhaustion in 1832 was followed by Maudleys' description of the same disorder in 1867 (inset). Agitated delirium signs and symptoms were reported in hyperactive or mixed forms of the disorder throughout the pre-neuroleptic era of psychiatry (Kraines, 1934; Stauder, 1934; Larson, 1939).

In 1934, Stauder published detailed observations of 27 cases, which became the definitive description of a syndrome that he termed lethal catatonia (Stauder, 1934). The cases were mostly young people, in the age range of 18–26 years, who had no significant premorbid psychological or physical disturbances. Stauder observed the acute onset of a severe form of psychomotor agitation that he called “elementary catatonic excitement.” Various degrees of clouding of consciousness and a strong tendency toward violent and self-destructive acts also were present. Although different nomenclature was used to describe a psychotic exhaustion syndrome, fatal cases of a life-threatening febrile neuropsychiatric disorder were widely recognized and reported by clinicians before modern psychiatric treatments became available (Shulack, 1946). The authors of these published reports found it remarkable that autopsies of these patients failed to reveal any clues to etiology or the cause of death, other than exhaustion.

Between 1954 and 1975, the advent of the neuroleptic drugs like Thorazine transformed psychiatric practice and reduced the incidence of exhaustive mania in institutionalized and unmedicated patients. However, the cocaine epidemic of the 1980's lead to a series of case reports describing sudden death in cocaine abusers with an extreme behavioral malady similar to what had been reported by Bell and others 150 years earlier. The agitated cocaine delirium deaths were associated with cocaine abuse and their appearance coincided with the introduction of cocaine into the United States (Fishbain and Wetli, 1981; Wetli, 1987). The trans-shipment of cocaine to South Florida through the Bahamian corridor and the increased incidence of cocaine-related medical emergency room admissions and drug related deaths placed Medical Examiners in Miami-Dade at the forefront of a new wave of cocaine-related excited delirium deaths.

Wetli and Fishbain (1985) described a case series of psychosis and sudden death in cocaine abusers, which was the first report of drug-related excited delirium (Table 1). The deaths occurred mostly in young cocaine intoxicated males, who exhibited extreme hyperactivity and violent behavior, hyperthermia and sudden cardiorespiratory collapse. Because these patients always presented with agitated and bizarre behavior, law enforcement was often called to the scene. The typical course was that after police restrained the individual, they died unexpectedly and suddenly following the use of various force methods, including maximal restraints, baton strikes, or use of noxious chemical “pepper” sprays (Wetli, 1987; Ross, 1998; Stratton et al., 2001). Medical examiner review of these cases did not reveal a definite anatomic cause of death, although drug overdose, trauma, and underlying cardiac disease were excluded (Wetli, 1987; Ruttenber et al., 1997; Stephens et al., 2004).

**Table 1:** **Historical descriptions and terminology of excited delirium syndrome**.

**Author and year**	**Nomenclature**	**Clinical description**
Calmeil, 1832	Delirious mania	Rare, life-threatening psychosis extreme hyperactivity, mounting fear, stuporous exhaustion
Bell, 1849	Bell's mania	Sudden onset of hyperactive arousal, confusion, transient hallucinations, core body temperature dysregulation, 75% mortality rate
Maudsley, 1867	Acute maniacal delirium	Violent mania, rapid pulse, constant motion, elevated temperature of skin, complete exhaustion
Stauder, 1934	Lethal catatonia	Intense motor excitement, violent, suicide attempts, intermittent rigidity, incoherent speech, bizarre delusions; fever (43.3°C), cardiovascular collapse
Wetli and Fishbain, 1985	Excited delirium	Agitation motor excitement, super human strength, paranoia, mounting fear, hyperthermia, cardiorespiratory collapse, cocaine intoxication, no anatomic cause of death

## Fatal cocaine delirium as a variant of the neuroleptic malignant syndrome

Neuroleptic malignant syndrome (NMS) is a rare, life-threatening idiosyncratic reaction to antipsychotic drugs characterized by fever, altered mental status, muscle rigidity, and autonomic dysfunction (Levenson, 1985; Weinberger and Kelly, 1977; Berman, 2011). The hallmark symptoms of NMS include hyperpyrexia and muscular rigidity, while the cocaine-associated syndrome is atypical in having minimal rigidity. Based on these similarities, Kosten and Kleber (1988) proposed that cocaine-induced excited delirium should be considered a dopamine agonist variant of NMS. Wetli (2005) proposed that NMS might be an attenuated form of acute exhaustive mania/excited delirium. These observations lead him to hypothesize that there may be three related syndromes: (1) acute exhaustive mania, as described by Bell in psychiatric patients, (2), excited delirium, due to psychostimulants; and (3) the attenuated variant—NMS (for review, Wetli, 2005).

Delirious mania and malignant catatonia both have non-malignant and malignant clinical features with early, non-malignant symptoms responding to neuroleptics, while patients who pass over into the malignant phase require sedation by benzodiazepines (Mann et al., 2013). Although NMS is a rare, life-threatening idiosyncratic reaction associated with virtually all neuroleptics, including the newer atypical antipsychotics (e.g., dopamine blockers), the condition is linked also to the use of indirect and direct-acting dopamine agonists. The abrupt cessation or reduction in dose of dopaminergic agonists, such as levodopa, pergolide, and amantadine in Parkinson's disease may precipitate NMS in vulnerable patients (Ito et al., 2001; Reimer et al., 2002). Interestingly, the akinetic crisis of Parkinson's disease is associated with a severe loss of striatal dopamine transporter function (Kasssinen et al., 2014). This rare condition is a life-threatening complication of Parkinson's disease, with an estimated annual incidence of 0.3% and death rate of 15%, that is associated with hyperthermia, dysautonomia, and increased serum muscle enzymes (Takubo et al., 2003; Onofrj et al., 2009). The clinical picture is similar to that of NMS and has been termed as the malignant syndrome of parkinsonism-hyperpyrexia. The condition is not related to disease stage or medication dosage, but one of the main features is that, the akinetic crisis appears to be long lasting (on average 11 days) and the dopamine system is transiently blocked from treatments, which would usually give patients rapid motor benefit. To date, none of the theories put forth as the underlying cause of the NMS related syndrome in Parkinson's disease have been able to explain why only a small fraction of patients exposed to dopaminergic agonists develop the condition, although state (dopaminergic drugs) and trait (genetic) vulnerabilities are likely risk factors.

Hypothalamic dopamine antagonism leads to the elevated set point for thermoregulation and the myotoxicity associated with malignant hyperthermia. Sympathoadrenal hyperactivity and the loss of hierarchical integration and homeostatic control may constitute important risk factors for NMS and its associated variants. Gurrera (1999) advanced this hypothesis, suggesting that sympathetic nervous system hyperactivity should be viewed as primary in the etiology of NMS. The sympathetic nervous system mediates the hypothalamic coordination of thermoregulatory activity and is a regulator of muscle tone and thermogenesis. The sympathetic nervous system's latent capacity for autonomous activity is expressed when tonic inhibitory inputs from higher central nervous system dopaminergic centers are disrupted. The predominant sources of spinal dopamine are the descending fibers projecting from the dopaminergic A10 and A11 cell groups of the posterior hypothalamus (Skagerberg and Lindvall, 1985; Qu et al., 2006). These tonic inhibitory inputs relay to preganglionic sympathetic neurons by way of the dopaminergic hypothalamospinal tracts.

A predisposition to more extreme sympathetic nervous system activation and/or dysfunction in response to emotional or psychological stress may be an underlying state vulnerability for NMS, as well as, for the ExDS associated with psychostimulant abuse. State variables like the acute psychic stress reported originally in Bell's mania when coupled with a loss of presynaptic dopaminergic transporter function may lead to extremely elevated concentrations of synaptic dopamine, and the emergence of related clinical syndromes.

## Excited delirium is a syndromal disorder of dysregulated dopamine

A syndrome is the association of several clinically recognizable features, signs, symptoms, or characteristics that often occur together, so that the presence of one feature alerts to the presence of the others. Most recognize that the condition of excited delirium represents a syndromal disorder rather than a specific disease. What has not been emphasized in the literature is that various organic brain disorders, as well as functional psychiatric conditions and psychostimulant abuse, contribute to the expression of a CNS disorder with high fatality rates that share a common underlying neurochemical dysregulation of central dopamine homeostasis.

Persons at risk for excited delirium are most likely at the extreme end of the neuropsychiatric continuum of several DSM-IV recognized disorders, including delirium induced by a drug, manic excitement, and psychomotor agitation (Vilke et al., 2012). Those at risk for excited delirium and sudden death include people who are withdrawing from or non-compliant with psychotropic drugs, substance abusers suffering from reward deficiency syndrome or alcoholics in withdrawal, and persons suffering from acute manic episodes that may be triggered or worsened by sleep deprivation.

The clinical description of excited delirium includes reports of increasing excitement with wild agitation and violent, often destructive behavior that can last for hours to days. The forensic pathology descriptions suggest that the disorder can wax and wane in severity over time with rigidity or stupor alternating with excitement (Wetli, 2005; DiMaio and DiMaio, 2006). These progress to increasing and possible fluctuations of fever and persistent autonomic instability with rapid and weak pulse and hypotension. Cocaine delirium shares clinical similarity to the acute onset of excitement, grandiosity, emotional lability, delusions, and insomnia associated with emergence of mania, and the disorientation and altered consciousness characteristic of delirium. Psychostimulant intoxication, drug withdrawal states, and undiagnosed mania and bipolar affective disorder are the most commonly reported antecedents (Wetli, 2005; Mash et al., 2009; Vilke et al., 2012).

## Pathophysiology and neurochemical triggers

Transmission of reward signals is a function of dopamine, a neurotransmitter known to be involved in the mechanism of psychosis. The symptoms of psychosis and mania are both related to dopaminergic hyperactivity in brain circuits implicated in neuropsychiatric disorders (Cipriani et al., 2011). In psychosis, post-synaptic receptor sensitization causes dysfunctional neural processing, leading to the development of delusional symptoms. This understanding fits well with the traditional hyperdopaminergic hypothesis of psychosis and schizophrenia. The hyperdopaminergia and disordered signaling in dopamine target regions of the brain also serves as a model for mania, since dopaminergic blocking drugs are effective in alleviating mania and psychosis.

Mania is the cardinal feature and a core symptom of bipolar disorder. PET scans in medicated, manic patients show abnormal brain activation in dorsal anterior cingulate, frontal polar, and right inferior frontal cortical regions (Rubinsztein et al., 2001). The increase in task-related anterior cingulate activation was positively correlated in this study with the severity of manic symptoms. Anterior cingulate cortex activation may be related to increased nucleus accumbens dopamine signaling, which leads to cortical and subcortical hyperactivity in mania (Perry et al., 2001). Genetic linkage studies have suggested an association of the dopamine transporter gene (Kelsoe et al., 1996; Greenwood et al., 2001, 2006) and lower levels of transporter protein expression in patients with bipolar affective disorder (Amsterdam and Newberg, 2007).

Cocaine and methamphetamine increase extracellular dopamine and produce behavioral effects similar to mania (Silverstone et al., 1983). Drug sensitization occurs in drug addiction, and is defined as an increased effect of a drug following repeated doses (the opposite of drug tolerance). Such sensitization involves increased brain mesolimbic dopamine transmission, as well as altered protein expression within mesolimbic dopamine neurons. Repeated treatment with psychostimulants leads to sensitization or reverse tolerance in animal models (Post and Rose, 1976; Hooks et al., 1994; Pierce and Kalivas, 1997; Zapata et al., 2003) and human cocaine abusers (Ujike and Sato, 2004; Seeman, 2011). Paranoia in the context of cocaine abuse is common and potentially dangerous and several lines of evidence suggest that this phenomenon may be related to loss of function of the dopamine transporter protein (Gelernter et al., 1994; van Dyck et al., 2005). These observations suggest that certain dopamine transporter genotypes might predispose to paranoia with chronic psychostimulant abuse.

The dopamine transporter undergoes neurobiological adaptations with chronic abuse of cocaine, depending on the duration, amount and pattern of use (e.g., binge vs. daily use). Intermittent cocaine self-administration in rodents produces sensitization of the stimulant effects of cocaine at the dopamine transporter (Calipari et al., 2014) and enhanced locomotor responsiveness or what is termed behavioral sensitization (Kalivas and Duffy, 1993; Robinson and Berridge, 1993; Kalivas et al., 1998). This phenomenon is not unique to cocaine; other psychomotor stimulants, some other classes of drugs, and mental stress induce the phenomenon of behavioral sensitization. Since cocaine directly inhibits dopamine reuptake by binding to the transporter, repeated cocaine administration may lead to a reduced potency of cocaine, which leads to an elevation in synaptic dopamine and the expression of behavioral sensitization (Zahniser et al., 1995, 1998).

**Figure 1:**
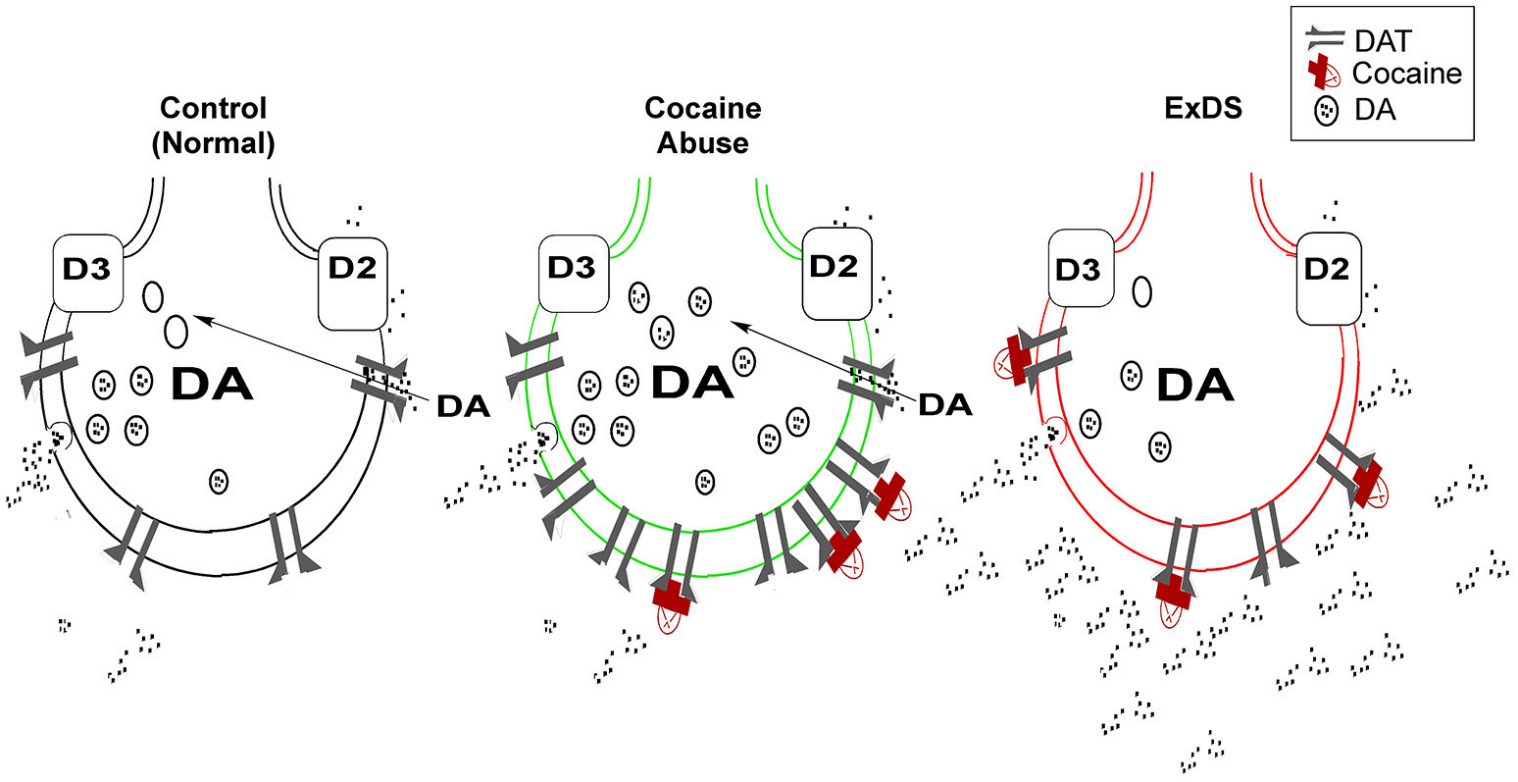
**Dysregulated dopamine transporter function in ExDS**. Located on presynaptic dopamine nerve terminals, the dopamine transporter functions to regulate the duration and intensity of synaptic dopamine signaling (left). Cocaine (red) inhibits the reuptake of dopamine by blocking the transporter protein (center). With chronic cocaine abuse, the dopamine transporter is trafficked to the plasma membrane as a compensatory adaptation to increases in synaptic dopamine. In ExDS victims, there is a loss of dopamine transporter regulation, which causes dopamine overflow in the synapse (right). The elevated synaptic dopamine leads to a state of hyperdopaminergia, that is associated with the intense motor excitement, paranoia, bizarre, and often violent behavior. DAT, dopamine transporter; DA, dopamine; D2, D2 dopamine receptor; D3, D3 dopamine receptor.

The dopamine transporter expressed in presynaptic terminals of dopamine neurons regulates reuptake of dopamine from the synaptic cleft and keeps extracellular dopamine concentrations low (Amara and Kuhar, 1993; Giros and Caron, 1993; Mortensen and Amara, 2003). The dopamine transporter is critical in regulating the concentration of extracellular dopamine and overall dopaminergic tone (Mash and Staley, 1996; Drevits et al., 1999; Mash et al., 2002, 2009). By blocking the transporter protein, cocaine allows released dopamine to persist in the extracellular space, which prolongs dopamine receptor stimulation (Figure 1). A decrease in dopamine transporter numbers or function in response to cocaine leads to reduced dopamine reuptake, elevated synaptic dopamine, and increased dopamine signaling at postsynaptic receptors.

The syndrome of excited delirium in drug abusers demonstrates that cocaine is the most frequent reported illicit drug (Ruttenber et al., 1997; Mash et al., 2009; Vilke et al., 2012). Most drug-related excited delirium victims are chronic freebase cocaine (“crack”) abusers, usually engaged in a “binge” pattern of drug use (Mash et al., 2002, 2009; Wetli, 2005). These persons use large amounts of “crack” cocaine or methamphetamine often for days, which interrupts normal sleep-wake cycles. Inhibition of dopamine transporter function is thought to be the primary mechanism underlying cocaine's addictive effects (Ritz et al., 1987). Although excited delirium is most frequently reported in cocaine abusers, psychostimulants including, methamphetamine, MDMA, alpha-PVP, methylome, and ephedrine have been associated with the syndrome (Mash et al., 2009; Penders et al., 2012). These psychostimulants directly interact with the dopamine transporter to cause a marked increase in the levels of synaptic dopamine.

Postmortem neurochemical studies of the human brain at autopsy demonstrate that chronic cocaine abuse leads to a compensatory upregulation of dopamine transporter number and function (Staley et al., 1994; Little et al., 1999; Mash et al., 2002). In contrast, there was no compensatory upregulation in dopamine transporter numbers in a case series of 90 cocaine-related excited delirium and exhaustive mania victims (Mash et al., 2009). The cocaine-related excited delirium cases occurred in persons who had reported histories of chronic cocaine abuse, consistent with the quantification of benzoylecgonine in blood and cocaine and benzoylecgonine measured in brain at autopsy (Mash et al., 2009). Mean core body temperature among the 90 victims was 40.7°C. Although the majority tested positive for cocaine, four had no licit or illicit drugs or alcohol measured in blood at autopsy. Forensic review of these four cases reported the cause of death as acute exhaustive mania, similar to the original description reported by Bell (1849).

All psychostimulants (e.g., cocaine, methamphetamine, and MDMA) increase the synaptic levels of dopamine (Amara and Kuhar, 1993; Giros and Caron, 1993), which may explain why chronic psychostimulant abusers are more at risk for exhibiting the behavioral symptoms associated with ExDS. A central role of dopamine is to mediate the “salience” of environmental events and internal representations in a dynamic process characterized by time and stimulus-dependent neural regulation (Kapur, 2003; Howes and Kapur, 2009). Dopamine can enhance both approach and avoidance behaviors and trigger extreme fear (Faure et al., 2008). In chronic cocaine abusers, there is a compensatory upregulation in dopamine transporter function, which is an adaptive increase to offset dopamine overflow in the synapse (Figure 1). When this homeostatic control of synaptic dopamine fails, it leads to a functional hyperdopaminergia, which triggers the acute onset of delirium and marked agitation in ExDS victims (Staley et al., 1996; Wetli et al., 1996; Mash et al., 2002, 2009).

Rhabdomyolysis secondary to mania and cocaine excited delirium is related to extreme physical exertion, although increased sympathetic tone during manic states and elevated epinephrine also play a role in its development (Manchip and Hurel, 1995; Ruttenber et al., 1999). Ruttenber et al. (1999) suggested that cocaine-associated rhabdomyolysis and excited delirium are components of the same syndrome and share the same initiating factors and pathophysiologic processes. Both hyperthermia and hyperactivity play important roles in the evolution of cocaine-associated rhabdomyolysis and excited delirium. Interestingly, in NMS, the elevated risk for hyperthermia results from disordered dopamine signaling precipitated by chronic administration of neuroleptic drugs (Strawn et al., 2007). The hyperthermia of neuroleptic malignant syndrome is associated with psychomotor agitation, and both syndromes have been related to increases in dopamine concentrations involved in thermoregulation and neuromuscular homeostasis (Keck et al., 1989).

Some undiagnosed psychiatric patients or those who are neuroleptic medication non-compliant may be at increased risk for excited delirium and sudden cardiac death. Dopamine transporter numbers fall below the normal homeostatic range for regulating dopamine in all cases of fatal excited delirium, including those with no known history of drug abuse and a negative toxicology screen at autopsy. These results suggest that the unabated conditions, which favor the development of excited delirium, are psychostimulant abuse, extreme mental stress or an underlying, or perhaps undiagnosed psychiatric condition.

A final common pathway for excited delirium related to chronic stimulant drug abuse, extreme environmental stress or acute mania of bipolar disorder might be a failure of the dopamine transporter to dynamically regulate synaptic dopamine. This failure of regulation leads to a hyperdopaminergic state, which triggers the violent behavior, delirium, agitation, and motor excitement. Dopamine systems in the brain also play a role in temperature regulation (Mann and Boger, 1978). The rise in core body temperature is most likely induced by dopamine stimulation of D1 receptors in the human hypothalamus which occurs because of a downregulation in D2 mediated hypothermia (Mash, 2009). A dopamine transporter murine model of hyperdopaminergia displays a distinctive cardiorespiratory and thermal phenotype, providing further support for altered dopamine transporter regulation in excited delirium (Vincent et al., 2007). Dopamine also regulates sleep and arousal, suggesting that there might be an inter-relationship between thermal behavior and circadian rhythms mediated by disrupted CNS dopamine signaling in excited delirium.

## When neurocardiac signals turn lethal

Mental and emotional stress is expressed in the brain as fluctuations in the activity of a subset of brain regions, including the insula, cingulate cortex, and amygdala (Critchley, 2009). These regions serve as an interface between emotional feeling states and visceral responses of the body. The insula and cingulate are viscerosensory cortices, which function to regulate attention and autonomic arousal. The amygdala is important in detecting and learning threat even in the absence of conscious awareness. The insula and cingulate cortices and subcortical regions of the limbic brain are heavily innervated by dopaminergic projections from the ventral tegmental nucleus (Gaspar, 1989). These closely connected brain regions together with the dorsal and ventral striatum are viewed as a “salience network,” acting directly on hypothalamic and brainstem centers to increase our bodily arousal state through direct coupling with sympathetic and parasympathetic efferent nuclei and feedback control loops located in the brainstem.

The insular cortex and the infralimbic cortex are part of a network involved in the descending control of the cardiovascular system (for review, Cechetto, 2013). These forebrain regions are responsible for integrating emotional and cognitive aspects related to cardiovascular responses. Together with the autonomic nervous system nuclei of the brainstem, these forebrain regions regulate cardiac function and electrophysiology via direct neural influences. Taggart et al. (2011) suggest that the roles of mental stress and emotion in arrhythmogenesis and sudden cardiac death are no longer confined to the realm of anecdote, but should be viewed as contributors to the pathophysiology of cardiac sudden death. Sympathetic arousal magnifies the electrophysiological effects of ischemia (for review, Taggart et al., 2011) and abnormal brain activity during seizures is associated with abnormalities of cardiac repolarization, and peri-ictal VT/VF in the absence of any visible cardiovascular disease (Taggart, 2013). A centrally triggered arrhythmia is a likely cause of sudden unexpected death in epilepsy (Surges et al., 2010), and a similar neurocardiac mechanism could underlie the sudden cardiac collapse in ExDS.

An emerging theme from animal models of asphyxia and cardiac arrest supports the notion that the autonomic nervous system is under constant surveillance by the cerebral cortex to ensure functional integrity of vital organs (Borjigin et al., 2013). A life-threatening crisis of the heart, with a rapid and steep change in heart rate and cardiac output, markedly activates and recruits the cerebral cortex to form a hierarchical circuit of cardiac survival. When transient homeostatic feedback from the brain to the heart is insufficient to restore cardiac function, the brain may exhibit a sustained activation that causes a premature and rapid death of the heart (Li et al., 2015).

Dopamine (DA) is an immediate precursor of noradrenaline that has stimulatory or inhibitory effects on a variety of adrenergic receptors. Dopamine also suppresses responsiveness to hypoxia, both in the carotid bodies and in the CNS (Huey et al., 2000). Although the physiological relevance is not established, one possibility is that suppression of stimulatory responsiveness to hypoxia is a protective mechanism directed against excessive discharge activity in the CNS respiratory network, which can result in neuronal excitotoxicity (Richter et al., 2000, for review Lalley, 2008). In the rat asphyxia model of cardiac sudden death, at as early as time 0, a sevenfold elevation in dopamine levels is measured by microdialysis with increased 3-MT (3-methoxytyramine) and decreased DOPAC (3,4-dihydroxyphenylacetic acid) concentrations. Since hyperdopaminergia is a condition of ExDS, the increase in synaptic dopamine may account for the decreased respiratory rate caused by dysfunctional adaptations in respiratory network rhythm.

Victims of ExDS usually die from cardiopulmonary arrest (Takeuchi et al., 2011; Vilke et al., 2012). Sudden cardiac death induced by a life-threatening stressor results from a generalized sympathetic storm within the autonomic nervous system (Samuels, 2007). Consistent with this view, exposure to carbon dioxide leads to an immediate systemic surge of neurally released dopamine and norepinephrine in asphyxic rats (Li et al., 2015). Experimental brain stimulation of the left insula can induce QT prolongation, bradycardia, and pulse-less asystole (Oppenheimer et al., 1991; for review, Taggart, 2013), similar to the sudden loss of vital signs with asystole reported in ExDS victims (Vilke et al., 2012). Life-threatening stress can lead to sudden cardiac death in people with no previous history of abnormal heart and brain function (Samuels, 2007; Sharkey et al., 2011). These observations suggest that autonomic toxicity induced by central hyperdopaminergic activity in ExDS may hasten the demise of heart function (Samuels, 2007; Li et al., 2015).

## Conclusions

Elevated synaptic dopamine when coupled with failed dopamine transporter function leads to agitation, paranoia and violent behaviors associated with ExDS. CNS dopamine also regulates heart rate, respiration, and core body temperature with chemical imbalance resulting in tachycardia, tachypnea, and hyperthermia. Hyperthermia is a hallmark of excited delirium and a harbinger of death in this syndromal disorder. Victims of excited delirium are in an extremely heightened emotional state exhibiting marked paranoia and mounting irrational fear. Abnormal signaling in the brain-heart axis may be a precipitant of a sudden fatal arrhythmia, since hyperdopaminergic signaling in the limbic system can convert extreme emotional stress into autonomic toxicity. The connection between the hyperdopaminergia and chaotic signaling in higher brain autonomic regulatory centers may explain the abrupt loss of autonomic function that leads to sudden unexpected death in victims of the ExDS.

Excited delirium is a syndromal disorder, which is controversial and highly debated precisely because the mechanism of lethality is unknown. However, molecular studies of the brain of autopsy victims who died in states of excited delirium reveal a loss of dopamine transporter function as a possible trigger of a lethal cascade of neural activities that progress to asphyxia and sudden cardiac arrest. Both national and regional ExDS registries are needed with data about medical history, toxicology, gender, and race to improve outcomes and further translational molecular research studies of this highly disputed and often unrecognized psychopathological condition associated with central dopamine dysfunction.

## Author contributions

The author confirms being the sole contributor of this work and approved it for publication.

## Funding

The original studies were funded by grants from the National Institute on Drug Abuse (NIDA) (DA06227; DA033684).

### Conflict of interest statement

The author declares that the research was conducted in the absence of any commercial or financial relationships that could be construed as a potential conflict of interest.
